# Full recovery of vision following early and intensive hemodialysis in an 18-year-old woman with methylmalonic acidemia-related optic neuropathy

**DOI:** 10.1016/j.ymgmr.2025.101279

**Published:** 2025-11-27

**Authors:** Alicia Guertin, Raoul Kanav Khanna, Marine Tardieu, Maud François, Hélène Blasco, Isabelle Benz de Bretagne, Jean-François Benoist, Adrien Bigot, Nathalie Tressel, François Maillot, François Labarthe

**Affiliations:** a CHRU Tours, Reference Center for Inborn Errors of Metabolism ToTeM, Hôpital Clocheville and hôpital Bretonneau, 49 boulevard Béranger, 37 044 Tours Cedex 1, France; b CHRU Tours, Department of Ophthalmology, Hôpital Bretonneau, 2 boulevard Tonnellé, 37044 Tours cedex9, France; c CHRU Tours, Service de néphrologie-HTA, dialyses et transplantation rénale, Hôpital Bretonneau, 2 boulevard Tonnellé, 37044 Tours cedex9, France; d Laboratoire de Biochimie métabolique, hôpital Bretonneau, CHRU, Tours, France; e Université de Tours, Inserm, iBrain, UMR 1253, 37000 Tours, France; f Department of Biochemistry, Hôpital Necker, AP-HP, and Université Paris-Saclay, Paris, France; g Université de Tours, Inserm, Nutrition, Croissance et Cancer, UMR 1069, 37000 Tours, France

**Keywords:** Optic neuropathy, Mitochondrial disease, Methylmalonyl-CoA mutase, Long-term complication

## Abstract

**Background:**

Methylmalonic acidemia (MMA) caused by complete or partial deficiency of the mitochondrial enzyme methylmalonyl-CoA mutase (mut^0^ or mut- enzymatic subtype, respectively) leads to the accumulation of toxic organic acids, causing severe organ dysfunction and life-threatening complications. Despite appropriate treatment, optic neuropathy has been reported as a vision-threatening complication of MMA, often resulting in debilitating sequelae.

**Case report:**

We present the case of an 18-year-old woman with isolated mut° MMA and with chronic kidney disease stage 3 who presented with a rapid decline in distance and near visual acuity in both eyes. Goldmann visual field perimetry revealed bilateral central relative scotomas with preserved peripheral vision. Visual evoked potentials were unstructured in both eyes, despite normal retinal imaging, indicating optic neuropathy. Metabolic testing revealed elevated levels of methylmalonic acid in both plasma and urine, and increased lactate levels in the plasma and cerebrospinal fluid. Based on the hypothesis of subacute toxicity from methylmalonic acid metabolites, intensive intermittent hemodialysis was initiated, which rapidly decreased the patient's plasma methylmalonic acid level. We also added an oral supplement of coenzyme Q10 and vitamin E. With this treatment, the patient's vision rapidly recovered, and visual function normalized five months later. No progression of optic neuropathy occurred in the eight years follow up after a combined liver/kidney transplant procedure.

**Conclusion:**

Optic neuropathy is a rare long-term complication of isolated MMA, presumably due to the chronic or subacute accumulation of toxic methylmalonic acid metabolites. This case report highlights the importance of early and intensive hemodialysis in achieving favorable visual outcomes by reducing toxic metabolite levels in both blood and central nervous system. In the long-term, liver or combined liver-kidney transplantation should be discussed.

## Highlights

• Optic neuropathy is a long-term complication of MMA.
• Optic neuropathy is related to the accumulation of toxic methylmalonic acid derivatives.
• Hemodialysis reduces these toxic metabolites and improves vision loss in MMA.

## 1 Introduction

Isolated methylmalonic acidemia (MMA) is a genetically heterogenous group of autosomal recessive inborn errors of metabolism mainly caused by a deficiency of the mitochondrial enzyme methylmalonyl-CoA mutase (OMIM#251000, EC 5.4.99.2) or defects in the synthesis of its cofactor 5’deoxyadenosylcobalamin. This enzyme mediates the cellular breakdown of branched-chain amino acids, odd-chain fatty acids and cholesterol, converting methylmalonyl-CoA to succinyl-CoA, which is an intermediate of the tricarboxylic acid cycle [1,2]. Loss of enzyme activity leads to the accumulation of toxic organic acids, causing severe organ dysfunction and life-threatening complications [3]. The disease is characterized by the accumulation of methylmalonic acid in all body tissues and fluids. Most patients present during the neonatal period or early infancy with acute episodes of ketoacidosis and neurological distress. The progression of the disease is marked by recurrent episodes of potentially life-threatening metabolic decompensation, which are usually triggered by concurrent infection and metabolic stress. Many patients develop long-term complications that conventional treatment does not effectively prevent [[1], [2], [3], [4], [5]]. These complications include short stature, global developmental delay and cognitive impairment, movement disorders, epilepsy, as well as progressive liver and kidney disease [[1], [2], [3]]. The mechanisms responsible for these long-term complications are not well understood, but the prevailing hypothesis is that mitochondrial impairment plays a major role in their physiopathology [3]. First studies focused on the direct toxicity of methylmalonic acid and derivative accumulation, and more recently new hypotheses were also suggested, such as altered anaplerosis of tricarboxylic acid cycle or posttranslational modifications related to aberrant hyperacylation of multiple metabolic pathways [[6], [7], [8], [9]]. Visual loss related to optic neuropathy has also been recognized as a complication of MMA [[10], [11], [12]]. Although rare, visual loss is often severe, with most patients experiencing debilitating sequelae despite appropriate treatment.

We present the case of a female patient with isolated MMA who experienced a rapid decline in bilateral vision at the age of 18 due to optic neuropathy, which was associated with high plasma methylmalonic acid levels. Based on the hypothesis of subacute toxicity from methylmalonic acid metabolites, we initiated intensive intermittent hemodialysis (HD), and the patient's vision returned to normal within a few months.

## 2 Case report

We describe the case of an 18-year-old woman diagnosed with severe MMA. She was the only child of non-consanguineous parents and was born at 35 weeks of gestation following an uneventful pregnancy. At 4 months of age, she was diagnosed with B12-unresponsive MMA during an acute episode of coma with lethargy, hypotonia and vomiting, associated with ketoacidosis and hyperammonemia. Metabolic testing revealed increased excretion of methylmalonic acid in the urine (15,800 μmol/mmol creatinine, normal range (NR) <10). The MMA diagnosis was subsequently assessed by a severely B12-unresponsive decreased methylmalonyl-CoA-mutase activity in cultured fibroblasts. Molecular analysis identified two missense pathogenic variants in the *MMUT* gene (c.473G > T and c.655 A > T, resulting in the protein changes p.Gly158Val and p.Asn219Tyr, respectively, and associated with mut^0^ phenotype by functional studies [13]). She was treated with a low-protein diet, supplemented with an essential amino acid mixture lacking methionine, threonine, valine and isoleucine, as well as daily supplementation with l-carnitine and metronidazole for 10 days per month. With this treatment, metabolic status progressively improved and remained well controlled, with only two admissions for mild acute metabolic crises occurring before the age of 18. She was also treated with growth hormone from the age of 6 to 15 years due to short stature, and with oestrogen and progesterone supplementation from the age of 13 to 15 years due to delayed puberty (menarche at the age of 16 years). From the age of 10, she progressively developed pyramidal signs, including hyperreflexia and spasticity, which were more pronounced in her lower limbs. A cerebral magnetic resonance imaging (MRI) performed at the age of 11 showed mild subcortical white matter lesions and an Arnold-Chiari type I malformation, which was likely unrelated to MMA. Brain lactate was undetectable using magnetic resonance spectroscopy. During adolescence, the patient was overweight and had a moderate intellectual disability, which caused her to experience some academic difficulties and require speech therapy. She developed progressive renal failure, with a decrease in measured chrome-EDTA clearance from 64 to 40 mL/min/1.73m^2^ between the ages of 11.8 and 17.4 years. She also developed liver fibrosis, which led to the consideration of a combined liver and kidney transplant from the age of 18 years (the course of the liver disease had previously been reported in a case series [14]). Plasma methylmalonic acid levels, an index of metabolic status, remained in the range 300–600 μmol/L between the ages of 7 and 14 years, increasing to around 1000 μmol/L after the age of 16 (see Table S1 in supplemental material).

**Fig. 1:**
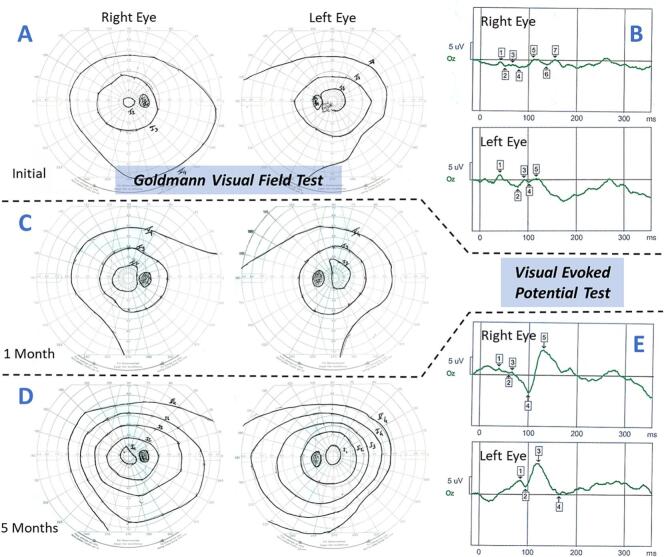
Follow-up of visual function. Goldmann visual field testing at admission (A), and after 1 month (C) and 5 months (D) of hemodialysis. (A) Reduced central sensitivity with absent I1 isopter, reduced amplitude of the I2 isopter, and preserved peripheral vision. (C) Gradual improvement at one month, with enlargement of the I2 isopter. (D) Normalization of the visual field with reappearance of the I1 isopter. Visual evoked potentials at admission (B) and after 5 months of hemodialysis (E); only the results from the 15-min arc checkerboard stimulus are presented. Visual evoked potentials were unstructured at admission (B) and showed significant improvement at 5 months (E), with reappearance of the P100 wave in both eyes. At 5 months, although latency of the P100 wave remained prolonged, amplitude was within normal limits in both eyes.

She was referred to our unit at the age of 18 because of a rapid decline in vision in both eyes over a period of 7 days. Ophthalmological examination confirmed bilateral vision loss, with distance best-corrected visual acuity (BCVA) of 20/100 in the right eye and 20/200 in the left eye. Near BCVA was also reduced, measuring Parinaud 4 in the right eye and Parinaud 8 in the left eye. Fundus examination and macular optical coherence tomography (OCT) were normal. Goldmann visual field testing demonstrated central sensitivity loss with preserved peripheral vision in both eyes (Fig. 1A). Visual evoked potentials (VEP, Fig. 1B) were unstructured in both eyes. Family history revealed no evidence of hereditary optic neuropathy, and plasma levels of vitamins A, E, B9 and B12 were within the normal range. Brain MRI showed no signs of ischemic or compressive lesions. Taken together, these findings were suggestive of optic neuropathy related to mitochondrial dysfunction in the papillomacular bundle.

A metabolic workup revealed elevated levels of methylmalonic acid in the plasma (2296 μmol/L, NR <0.4) and in the urine (8137 μmol/mmol of creatinine, NR <15), as well as increased lactate levels in plasma (3.7 mmol/L, NR <2.0) and concomitantly in CSF (7.22 mmol/L, NR <1.9). There was also a slight increase in transaminase levels in the blood (between 1.5 and 2 NR). The plasma creatinine level confirmed moderate renal failure, with an estimated glomerular filtration rate (eGFR) of 44 mL/min/1,73m^2^. Plasma ammonium and homocysteine levels were also within the normal range. Blood tests for infectious diseases were normal. We concluded that the optic neuropathy was possibly related to the toxic accumulation of methylmalonic acid and its derivatives in the central nervous system (CNS) and was exacerbated by renal failure, which resulted in decreased urinary excretion of these toxic metabolites.

An intensive HD program was started, with six 5-h sessions per week for three weeks. A few days after admission, oral supplementation of 200 mg/day of ubidecarenone and 200 mg/day of vitamin E was initiated because these treatments attenuate the renal disease of MMA in animal models and have been proposed in the treatment of MMA optic neuropathy [1,6,10,11,[15], [16], [17]].

**Fig. 2:**
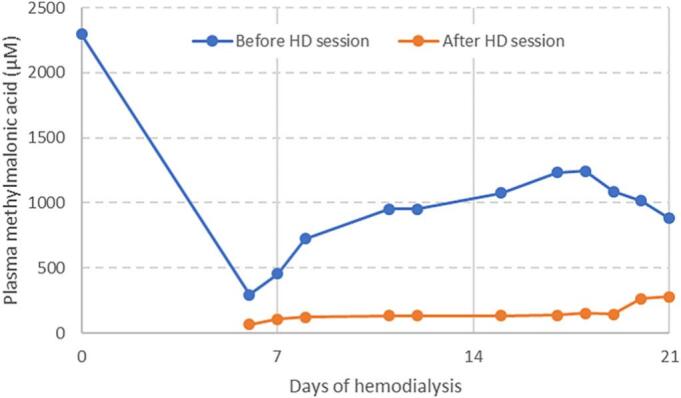
Follow up of plasma levels of methylmalonic acid during 3 weeks of hemodialysis. Results are expressed in μmol/L, before (blue line) or after (orange line) hemodialysis sessions. Normal value <0.4 μmol/L. Hemodialysis was performed intensively with 6 sessions per week, with a duration of 5 h for each session.

Fig. 2 shows the evolution of plasma methylmalonic acid levels following treatment. Plasma methylmalonic acid levels decreased in the range 65–280 μmol/L at the end of the HD session and increased in the range 300–1250 μmol/L before the next session. Plasma lactate levels remained mildly elevated, ranging from 3 to 4 mmol/L. In contrast, CSF lactate levels decreased from 7.22 mmol/L at admission to 3.83 mmol/L three weeks later.

One week after initiation of HD, the patient reported visual improvement. Fifteen days after iniating HD, distance BCVA improved to 20/80 in the right eye and 20/63 in the left eye, with near BCVA also improving to Parinaud 2 in both eyes. One month later, Goldmann visual field testing showed significant improvement in central sensitivity in both eyes (Fig. 1C). Five months later, BCVA returned to normal (i.e. 20/20 Parinaud 2) and Goldmann visual field testing showed full visual fields in both eyes (Fig. 1D). The VEPs showed significant improvement with the reappearance of the P100 wave, which had normal amplitude but prolonged latency (Fig. 1E).

After discharged, the patient continued HD three times per week, along with conventional metabolic management of MMA. Eighteen months later, a combined liver and kidney transplantation was performed. There was no recurrence of vision loss during this entire period, nor during the following eight years.

## 3 Discussion

We report the first case of a MMA patient with visual loss related to optic neuropathy who completely recovered after intensive intermittent HD.

MMAs are rare inherited metabolic diseases with multiorgan involvement. Despite the improvement of medical management, long-term prognosis is generally poor in severe forms mut°, both in terms of mortality and morbidity [[1], [2], [3]]. Chronic kidney disease is a common complication of MMA, leading to kidney failure, dialysis, and kidney transplantation [1,18]. Other long-term complications include various neurological symptoms (metabolic strokes and basal ganglia lesions, developmental delay, epilepsy, movement disorders, etc.), cardiomyopathy, pancreatitis, and liver abnormalities [[1], [2], [3],^14^]. Optic neuropathy-related vision loss is increasingly recognized as part of the long-term complications of MMA, primarily through isolated case reports or small case series. To our knowledge, twenty-three patients have already been reported in the literature [[10], [11], [12],^16^,^17^,^19^,^20^]. A detailed description of these patients and their long-term outcomes is presented in **Supplemental material 2**, which also includes data from our patient. Briefly, the patients had a median age of 15 years [range 6–28] and were evenly distributed between males and females. All had severe MMA. The median time between the onset of optic neuropathy symptoms and diagnosis was two months [range 0.2–24.0]. Diagnosing optic neuropathy in patients with MMA can be challenging due to developmental delay and the difficulty of performing visual tests that require patient cooperation, particularly in children. Available data suggest that VEPs play an important role, along with visual field testing and optical coherence tomography, when feasible. Brain MRI can provide supportive evidence of optic nerve involvement by showing hypersignals in the optic nerves, although such findings have been reported in only three patients and may reflect nonspecific optic atrophy. Based on the mitochondrial toxicity pathophysiological hypothesis, several patients were treated with coenzyme Q10 and/or vitamin E. While one patient showed only partial recovery with treatment, most patients did not experience any improvement in visual function. One patient experienced spontaneous partial improvement, and only one other patient benefited from HD and antioxidant treatment, also showing partial improvement. Our patient was the only one to have a complete recovery, with HD performed earlier and more efficiently than in the other patients treated with HD.

In our patient, vision loss was attributed to optic neuropathy, given the absence of evidence for alternative diagnoses such as ischemic, inflammatory, or compressive lesions; vitamin deficiency; or family history of optic neuropathy. In the context of MMA, the optic neuropathy was likely related to the underlying metabolic disorder, with a progressive and permanent increase in plasma methylmalonic acid levels. We hypothesize that the accumulation of methylmalonic acid and its derivatives in the CNS can be particularly toxic to optic nerves. The high energy demand of the papillomacular bundle, due to its lack of myelin, is associated with a higher concentration of mitochondria in the retinal ganglion cells compared to the peripheral retina. It is well established that the papillomacular bundle is particularly vulnerable to mitochondrial dysfunction [21]. Accordingly, our patient's phenotype, including central vision loss with preserved peripheral vision, is consistent with other mitochondrial optic neuropathies, such as Leber's hereditary optic neuropathy.

We hypothesize that mitochondrial dysfunction is related to toxic accumulation, as reflected by high plasma levels of methylmalonic acid, and exacerbated by decreased urinary excretion due to renal failure. A more marked increase in lactate in CSF than in blood could suggest more severe mitochondrial dysfunction in the CNS, including the optic nerves. Moreover, the decrease of CSF lactate following HD, reduction in plasma methylmalonic acid levels and improvement in vision, also suggest an improvement in energy production.

The mechanisms responsible for long-term complications in MMA remain poorly understood. All of them are presumably due to the accumulation of toxic metabolites of methylmalonic acid. More in details, methylmalonyl-CoA mutase deficiency leads to the accumulation of toxic organic acids (e.g., methylmalonic acid, propionic acid and 2-methylcitric acid) within the mitochondrial matrix, triggering structural and functional abnormalities in the mitochondrial network that drive severe organ dysfunction affecting primarily the brain, liver and kidney [6,[22], [23], [24], [25]]. Deficient energy metabolism has long been suspected to play a major role in MMA [23]. Early investigations suggested a mitochondrial dysfunction with a markedly decrease of cytochrome *c* oxidase activity in the liver and megamitochondria formation in the kidney proximal tubules in concert with electron transport chain dysfunction [6,9,26]. Numerous studies in vitro demonstrated that methylmalonic acid inhibits mitochondrial respiration in liver and brain [7,8]. Further investigations also demonstrated an indirect toxicity of methylmalonic acid and derivatives with an altered anaplerosis of tricarboxylic acid cycle and ammonium accumulation in blood and CNS [7,27]. However, the direct causative role of methylmalonic acid accumulation in optic neuropathy may be questioned because optic neuropathy has been reported in cases of minor methylmalonic acid accumulation, such as in B12-responsive cblA patients and after liver transplantation [28]. Additionally, optic neuropathy has been reported in propionic acidemia, in which the biochemical defect occurs upstream of MMA and the accumulated neurotoxic substrates differ from methylmalonic acid, suggesting another common pathophysiological mechanism [3,10,11]. More recently, mitophagy dysfunctions that trigger epithelial stress and ultimately cell damage, and aberrant hyperacylation of multiple metabolic pathways have been suspected to contribute to the physiopathology of long-term MMA complications [7,25]. For optic neuropathy, mitochondrial impairment seems to play a major role [3]. The age at onset, the presentation and the progression of disease mimics the clinical profile of Leber hereditary optic neuropathy and other mitochondrial optic neuropathies. Moreover, the beneficial effect on vision of coenzyme Q10 and vitamin E are classically proposed as neuroprotective agent in patients suffering from mitochondrial optic neuropathies [3,6,15,16,29]. Regardless of the underlying pathophysiological mechanisms, additional metabolic markers are necessary to evaluate disease severity and monitor disease progression. From this perspective, markers of mitochondrial dysfunction, such as fibroblast growth factor 21 (FGF-21) and growth differentiation factor 15 (GDF-15), could be promising prognostic indicators of organic acidemia complications associated with mitochondrial dysfunction [30].

In the aim of decreasing rapidly and consistently toxic derivatives, we initiated an intensive HD program that demonstrated a sustained decrease in plasma methylmalonic acid levels and a clinical improvement of vision in several months. Methylmalonic acid has a low molecular weight and is therefore easily cleared from plasma by diffusion on HD. However, plasma methylmalonic acid is continuously produced by the body. Thus, the rate of plasma methylmalonic acid production exceeds the capacity of intermittent HD to clear it, and its plasma level rebounds despite efficient dialytic clearance [20]. Moreover, there is often a gradient between serum and CSF methylmalonic acid, and it is possible that there is less improvement in the CSF methylmalonic acid levels than in the periphery [20,31,32]. Based on expert opinions, we proposed an intensive intermittent HD program, with 5 h per session and with 6 sessions per week, adapted to a precise monitoring of pre- and post-dialysis plasma methylmalonic acid levels [18,20]. On the long term, organ transplantation represents a form of partial enzyme replacement to improve the course of MMA [6]. There is evidence that early liver transplant greatly improves metabolic stability and reduces the risk of long-term complications [5]. For MMA, early liver transplant reduces methylmalonic acid levels which in turns reduces its effects on kidneys and therefore slows progression of chronic kidney disease [4,5]. At long-term, the combined liver and kidney transplantation improves patients' quality of life [33].

We recognize some limitations with this work. Firstly, the full recovery of visual acuity is very encouraging, but intensive HD treatment was started very early. It cannot be ruled out that such treatment initiated later in the course of the disease is only partially effective [20]. However, none of the patients previously described with optic neuropathy benefited from emergency HD. Secondly, our patient also benefited from neuroprotective treatment with coenzyme Q10 and vitamin E. It is possible that this treatment contributed to the improvement in vision. However, the same treatment was used in most patients with MMA and optic neuropathy but brought modest improvement in only one patient [16]. Finally, another patient experienced a spontaneous partial recovery of vision four months later, suggesting that such improvement is possible [11].

In summary, optic neuropathy is a rare complication of long-term isolated MMA. Its mechanism remains unclear but there is evidence in our case as in the literature that accumulation of toxic metabolites of methylmalonic acid plays a central role in the physiopathology of this complication. In case of vision loss associated with high plasma levels methylmalonic acid, we suggest an early and intensive HD aimed to rapidly decrease toxic metabolite levels in the blood and the CNS. The use of neuroprotective treatments such as coenzyme Q10 and vitamin E is also recommended. At long-term, liver or combined liver/kidney transplantation seems to be a good option to prevent neurological complications in patients suffering from severe isolated MMA.

## CRediT authorship contribution statement

**Alicia Guertin:** Writing – original draft, Investigation, Formal analysis, Data curation. **Raoul Kanav Khanna:** Writing – review & editing, Formal analysis. **Marine Tardieu:** Writing – review & editing, Data curation. **Maud François:** Writing – review & editing, Formal analysis, Data curation. **Hélène Blasco:** Writing – review & editing, Formal analysis. **Isabelle Benz de Bretagne:** Writing – review & editing. **Jean-François Benoist:** Writing – review & editing, Formal analysis. **Adrien Bigot:** Writing – review & editing, Formal analysis. **Nathalie Tressel:** Writing – review & editing, Investigation, Data curation. **François Maillot:** Writing – review & editing, Investigation, Formal analysis. **François Labarthe:** Writing – review & editing, Writing – original draft, Investigation, Formal analysis, Data curation, Conceptualization.

## Funding sources

This research did not receive any specific grant from funding agencies in the public, commercial, or not-for-profit sectors.

## Declaration of competing interest

FL reports consulting fees from PTC Therapeutics and Sanofi Genzyme, outside the submitted work. FM reports consulting fees from PTC Therapeutics, Otsuka, Travere therapeutics and Vitaflo. The other authors report no conflict of interest.

## Data Availability

Data will be made available on request.

## Appendix A Supplementary data

Supplementary material Image 1
